# Analysis of training pathway to reach expert performance levels based on proficiency-based progression in robotic-assisted minimally invasive esophagectomy (RAMIE)

**DOI:** 10.1007/s00464-023-10308-2

**Published:** 2023-08-14

**Authors:** Dolores T. Müller, Stefanie Brunner, Jennifer Straatman, Benjamin Babic, Jennifer A. Eckhoff, Alissa Reisewitz, Christian Storms, Lars M. Schiffmann, Thomas Schmidt, Wolfgang Schröder, Christiane J. Bruns, Hans F. Fuchs

**Affiliations:** 1 Department for General, Visceral, Cancer and Transplant Surgery, Faculty of Medicine and University Hospital Cologne, University of Cologne, Kerpener Str. 62, 50937 Cologne, Germany; 2 Afdeling Heelkunde, Amsterdam Universitair Medisch Centrum, Amsterdam, The Netherlands; 3 Center for Esophagogastric Cancer Surgery, St. Elisabethen Hospital Frankfurt, Frankfurt, Germany

**Keywords:** Robotic-assisted minimally invasive esophagectomy, RAMIE, Learning curve, Proficiency-based progression, PBP

## Abstract

**Background:**

Robotic-assisted minimally invasive esophagectomy (RAMIE) was first introduced in 2003 and has since then shown to significantly improve the postoperative course. Previous studies have shown that a structured training pathway based on proficiency-based progression using individual skill levels as measures of reach of competence can enhance surgical performance. The aim of this study was to evaluate and help understand our pathway to reach surgical expert levels using a proficiency-based approach introducing RAMIE at our German high-volume center.

**Methods:**

All patients undergoing RAMIE performed by two experienced surgeons for esophageal cancer since the introduction of the robotic technique in 2017 was included in this analysis. Intraoperative outcomes and postoperative outcomes were included in the analysis. The cumulative sum method was used to analyze how many cases are needed to reach expert levels for different performance characteristics and skill sets during robotic-assisted minimally invasive esophagectomy.

**Results:**

From 06/2017 to 03/2022, a total of 154 patients underwent RAMIE at our facility and were included in the analysis. An advancement in performance level was observed for total operating time after 70 cases and for thoracic operative time after 79 cases. Lymph node yield showed an increase up until case 60 in the CUSUM analysis. Length of hospital stay stabilized after case 55. The CCI score inflection point was at case 55 in both CUSUM and regression analyses. Anastomotic leak rate stabilized at case 38 and showed another inflection point after case 83.

**Conclusion:**

Our data and analysis showed the progression from proficient to expert performance levels during the implementation of RAMIE at a European high-volume center. Further analysis of surgeons, especially with a different training status has yet to reveal if the caseloads found in this study are universally applicable. However, skill acquisition and respective measures of such are diverse and as a great range of number of cases was observed, we believe that the learning curve and ascent in performance levels cannot be defined by one parameter alone.

Survival rates for patients with locally advanced esophageal cancer are—despite all technical and pharmaceutical advancements of the last years—still low [1]. For locally advanced but yet resectable tumors, a transthoracic esophagectomy with a 2-field lymph node dissection in a multimodal setting depicts the current standard and has shown to improve patients survival, with 5-year survival rates up to 40%. A three-field lymphadenectomy may be indicated if the tumor is located in the proximal thoracic esophagus [2, 3]. With the introduction of robotic devices and the routine application of minimally invasive techniques to every-day clinical practice, patient outcomes and oncological quality of surgery have been improved significantly compared to an open approach [4–6]. RAMIE was first introduced in 2003 and has since then shown to minimize operative trauma, lead to reduced pulmonary complications postoperatively, shorten length of hospital stay, and enhance quality of life [5, 7, 8]. However, the implementation of any new device, especially a complex surgical robotic device, is usually associated with a long learning curve. The term learning curve is defined as the process of gaining knowledge, improving skills, and reaching proficiency in performing a surgical procedure and may provide a parameter to evaluate the technical skill and benchmarks of surgical techniques while achieving competence [9]. A recent systematic review from Chan et al. exploring the learning curves of minimally invasive and robotic minimally invasive esophagectomy displays how analysis of learning curves is not standardized, nor are the included parameters which are defined as measures contributing to the skill acquisition [10]. Another meta-analysis from Pickering et al. was able to show that out of 15 studies depicting the learning curve of RAMIE, only two centers used a structured training pathway when first introducing the new technique. Previous studies have shown that a structured training pathway based on proficiency-based progression using individual skill levels as measures of reach of competence can significantly improve the surgical training [11, 12]. The aim of this study was to evaluate and help understand our pathway to reach expert levels of performance using a proficiency-based approach when first introducing RAMIE at our German high-volume center.

## Materials and methods

An analysis of our prospectively collected, IRB-approved database of RAMIE was performed. All patients undergoing RAMIE performed by two experienced surgeons for esophageal cancer since the introduction of the robotic technique in 2017 at our clinic were included in this analysis. Starting in January 2019, we implemented an updated robotic standardized anastomotic technique using a circular stapler and indocyanine green (ICG) for assessment of vascularization of the gastric conduit, for our RAMIE cases at our academic center. We have previously published a detailed report of our standardized oncological pathway [13, 14]. Our standardized surgical technique as well as our novel approach using ICG for lymphatic mapping for esophageal cancer (*The ESOMAP Trial*) have also been published [13, 15]. The current standard at our clinic depicts a laparoscopic approach for the abdominal part, combined with a robotic-assisted approach for the thoracic part. Patients were also included in the analysis if a totally robotic approach was chosen. Exclusion criteria included concomitant procedures performed in addition to RAMIE and a two-staged approach.

Data were retrospectively analyzed from a prospectively maintained database. The study was conducted in accordance with the Declaration of Helsinki; ethical review and approval were waived for this study due to the retrospective design of the study.

Duration of surgery was defined as incision-suture time in minutes. The abdominal part of the procedure was defined as the gastric mobilization including repositioning of the patient, the thoracic part included the esophagectomy, and reconstruction using the gastric conduit was defined as time of single lung ventilation. Postoperative complications were defined using the standard established by the ECCG (Esophagectomy Complications Consensus Group) and classified according to the Clavien-Dindo classification [16, 17]. Postoperative complications were further evaluated using the comprehensive complication index (CCI) [18]. Oncological quality of the resection was evaluated using the R0 resection rate and number of resected lymph nodes, number of positive lymph nodes, and corresponding nodal status. The specimen was sent to pathology “en bloc.” If additional lymph nodes were resected, a separate labeled container was sent in addition. Lymph nodes were then counted by the pathologist during histopathological analysis of the specimen and hematoxylin and eosin staining was performed for further workup.

Regarding ICU stay, our protocol includes the use of goal directed fluid therapy, immediate extubation after surgery, and the use of epidural anesthesia for pain management. Criteria for discharging a patient from ICU were the following:

- Patient is awake and alert, EMV max
- Free airway, oxygenation < 3 l O_2_ via nasal canula.
- Mean arterial pressure (MAP) > 65, without catecholamines.
- Urinary production > 30 ml/min,
- Electrolytes within range: natrium 135–145 mmol/l, Kalium 3.5–5 mmol/l
- X-ray thorax: shows adequate position of thoracic drain and gastric tube, no pneumothorax

Criteria for discharging a patient for the hospital included the patient's daily need of calories can be met by oral nutrition or at least liquid nutrition in combination with additional enteral nutrition via a jejunal feeding catheter, bowel movements are regular and adequate, the patient does not require oxygen during mobilization (short distances or stair climbing) or at rest. Sufficient analgesia obtained at rest and under mobilization with opioids and non-opioid analgesics, presenting with normal and stable vital signs unless already deviating from normal preoperatively, presenting with regressive infection parameters (leukocyte count < 12 G/l, CRP ≤ 80 mg/gl, starting from a stable and clinically appropriate context) and sufficient support in the home environment after discharge is provided (family members, outpatient care services, follow-up treatment).

### Cumulative sum method

The cumulative sum method (CUSUM) was used for further analysis. Intraoperative outcomes (operative time, estimated blood loss, conversion rate) and postoperative outcomes (CCI, anastomotic leakage, R0 resection rate, lymph node harvest, length of stay on intensive care unit, length of hospital stay) were included in the analysis. In order to perform CUSUM analysis, the observed measurement (e.g., operating time) for each consecutive case was compared to the cumulative mean, as a parameter for the expected outcome. The difference is cumulatively added to the preceding value. For example, when looking at operating time, if the operating time is longer than the mean time, the CUSUM increases, representing the learning curve. As soon as the operating time becomes lower than the mean, the CUSUM decreases as operating times decrease and the CUSUM graph trends downwards indicating the learning curve is overcome. CUSUM plots were generated for each of the abovementioned variables. Fitted locally estimated scatterplot smoothing (LOES) curves using generalized additive models were calculated and depicted for each learning curve variable. A visual assessment of inflection point was used to judge the point at which the learning curve had been overcome and mean operating times began to reduce.

### Proficiency-based progression and adult skill acquisition

**Fig. 1:**
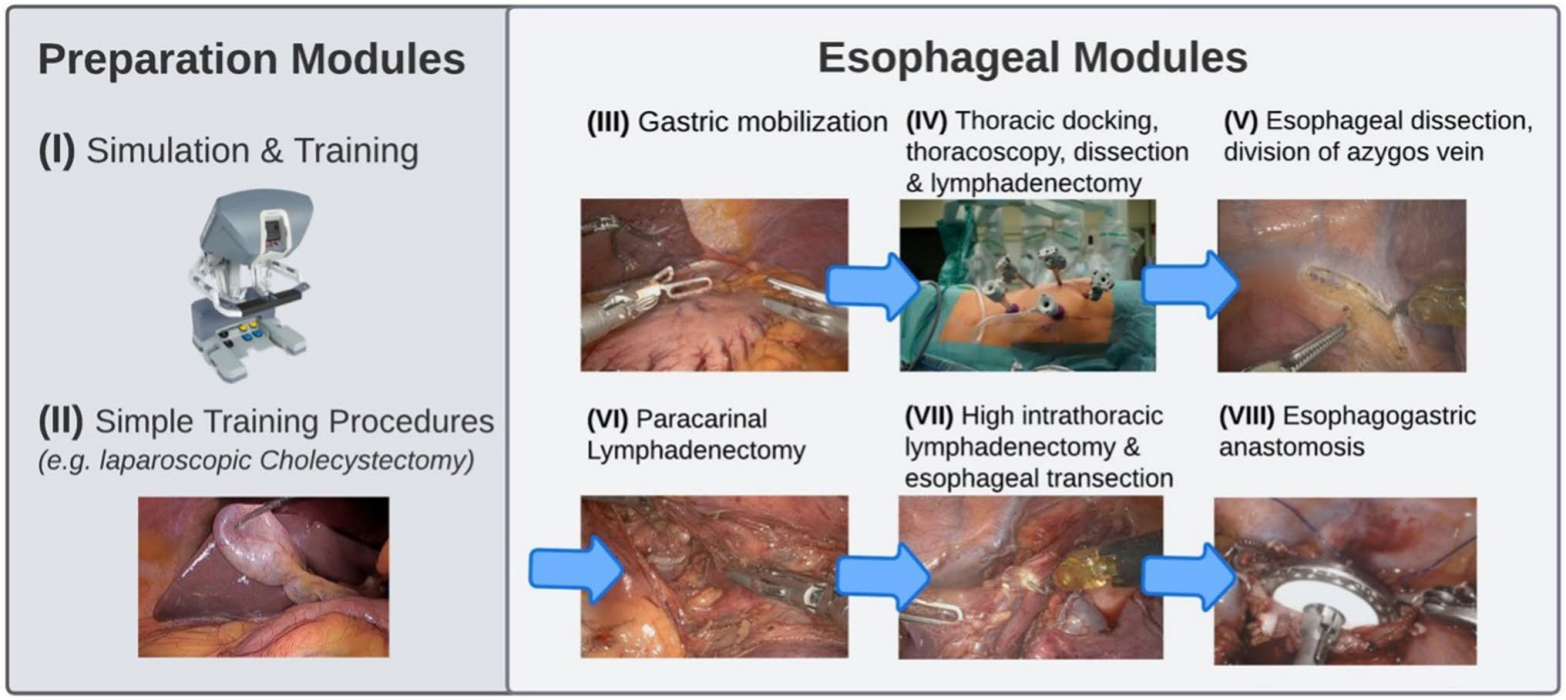
The modular step-up approach includes two preparation modules including simulation, inanimate and animate training, and simple training procedures followed by six manageable and straightforward esophageal modules

The introduction of a new surgical device often accounts for the simultaneous introduction of a new approach and often even a new surgical technique to ensure feasibility and patient safety. When we first introduced the da Vinci Xi surgical system (*Intuitive Surgical Inc, Sunnyvale, CA, USA*) to our center, a modular step-up approach was chosen [13]. This approach consisted of two prep modules including simulation, inanimate and animate training, and simple training procedures with increasing difficulties (i.e., cholecystectomy). Following these prep modules, the complex RAMIE procedure was divided into manageable and straightforward modules which are shown in Fig. 1. Moving forward, modules were completed based on achieving proficiency without compromising patient safety or surgical quality.

A previous worldwide Delphi consensus study initiated by our group has shown that there is a need for a structured training curriculum for robotic-assisted esophagectomy with 93% of participants agreeing that robotic training should be based on proficiency-based progression with benchmarking of pass/fail levels [19]. Proficiency-based progression (PBP) is an effective training algorithm that uses performance levels of individuals and different skill characteristics to ensure sufficient reach of competency and expert levels in contrast to conservative training techniques which usually use benchmarking and abstract performance metrics [20]. Advantages of PBP include the acquisition of a heterogenous and therefore broad skill set in combination with an individualized achievement of competency without a pre-defined number of training cases or time spent in training, ultimately putting the onus on the trainee. Previous studies have shown trainees to progress faster with improved and faster achievement of competency even when learning complex surgical tasks when using a PBP-based training program [11].

**Fig. 2:**
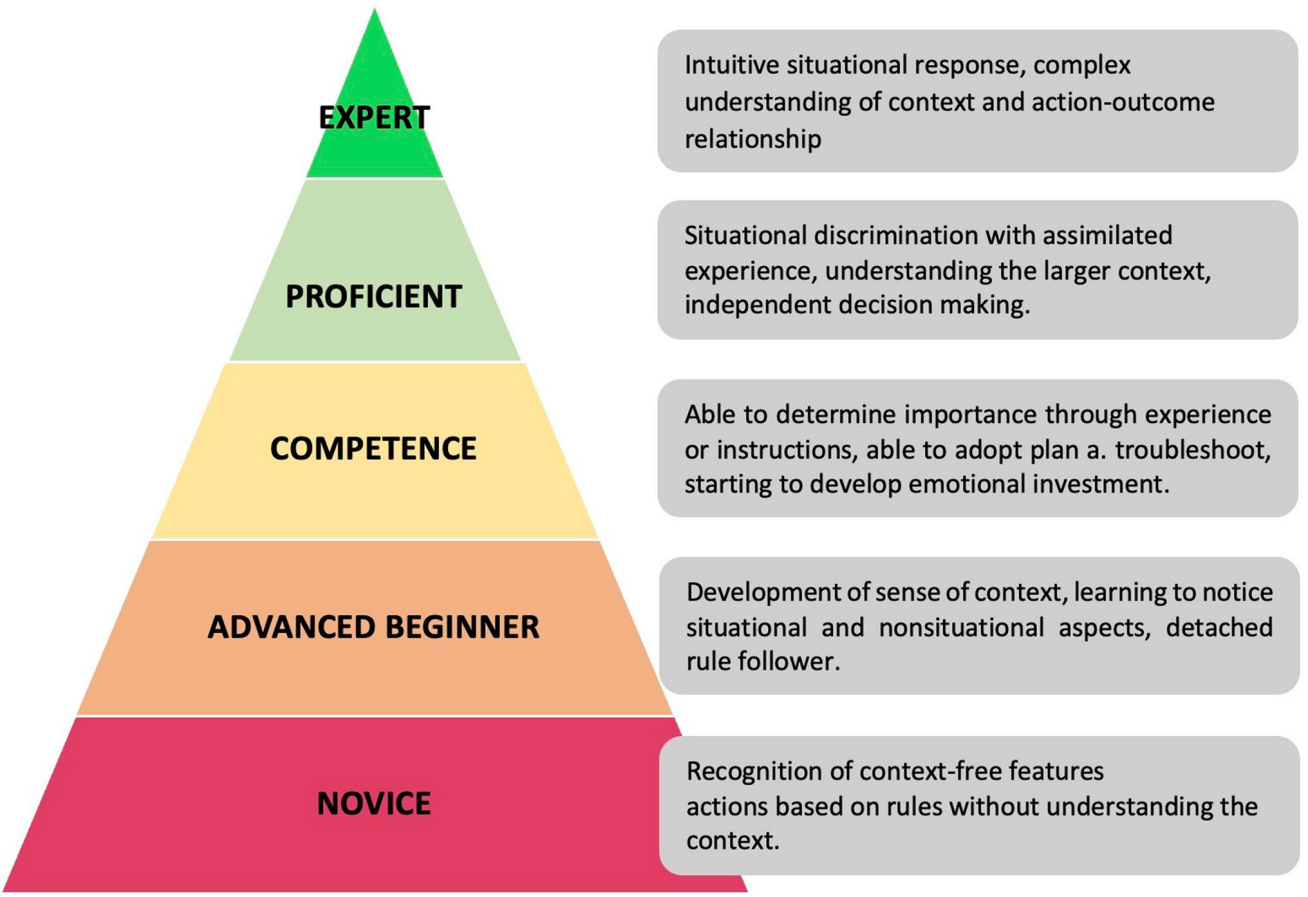
Adapted from Dreyfus & Dreyfus’ definition of adult skill acquisition [21]

**Fig. 3:**
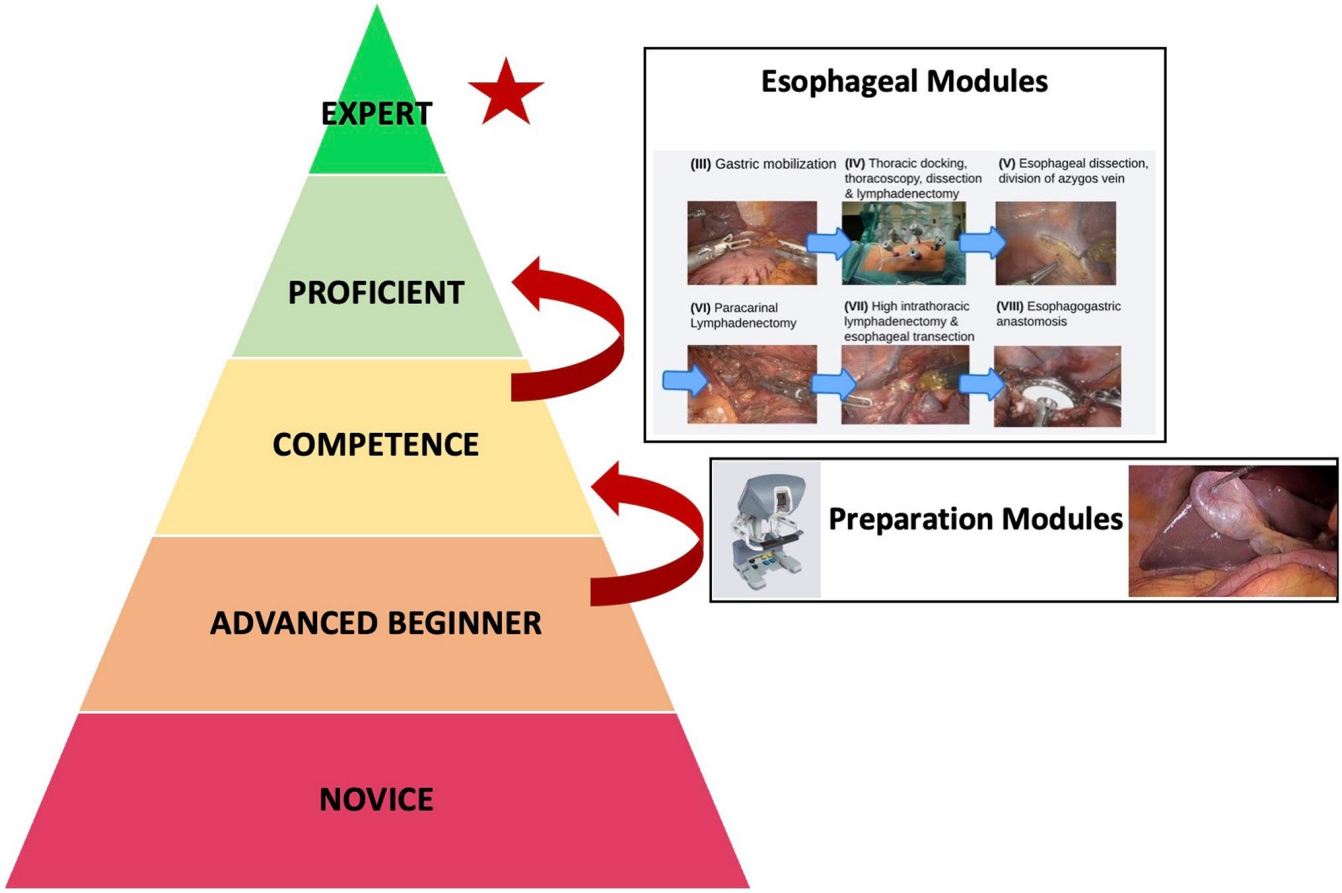
We started our robotic learning pathway as advanced beginners and were able to reach competency by completing preparation modules. Using a modular step-up approach, proficiency was achieved one module at a time until all modules were completed and a fully robotic-assisted minimally invasive esophagectomy was performed at a proficient performance level

Based on Dreyfus & Dreyfus’ definition of adult skill acquisition, we started our robotic learning curve for robotic-assisted esophagectomy as advanced beginners (Fig. 2) [20, 21]. Using prep modules, we were able to reach competency in robotic surgery and develop a conceptual model using defined modules as described above. Using a modular step-up approach, proficiency was achieved one module at a time until all modules were completed and a fully robotic-assisted esophagectomy was performed at a proficient performance level. The introduction of a standardized method using a circular stapling device and ICG for assessment of vascularization marks the reach of proficiency at our clinic. Figure 3 depicts a summary of our training pathway to reach expert performance levels based on PBP and the adult skill acquisition model from Dreyfus & Dreyfus [20, 21]. Benchmarks for postoperative outcomes after esophagectomy have been published before by the Esodata study group. [22] Hence, a proficient level of performance could be defined as keeping quality measures below published benchmarks. The cutoff for an expert level was then evaluated using those cases, where outcomes were below benchmarks and in line with the other established surgical approaches and assessment using the cumulative sum method was performed to define when the next performance level was reached.

### Statistical analysis

Statistical comparison and analysis were performed using Fisher’s exact test for nominal data and Student’s *t*-test for continuous data. A *p*-value of less than 0.05 was considered statistically significant. Continuous variables are presented as means and range. Categorical data are presented as numbers and percentages. Data were analyzed using GraphPad Software (San Diego, California, USA). Furthermore, the cumulative sum method was used to analyze how many cases are needed to reach expert levels for different performance characteristics and skill sets during robotic-assisted esophagectomy.

## Results

### Baseline and oncological characteristics

From June 2017 to March 2022, a total of 165 patients underwent RAMIE at our clinic. Of these, a total of 154 were included in the analysis. Eleven patients were excluded from the analysis as concomitant surgery was performed or a two-stage approach was chosen. Baseline characteristics of *n* = 154 patients are shown in Table 1. Most patients were male, with 80.5% (*n* = 124). Mean BMI was 25.5 kg/m^2^ (range 15.6–42.2 kg/m^2^). Mean age was 62.5 years (range 36–82 years). A mean of 37.2 lymph nodes were resected (range 18–97 lymph nodes).

**Table 1:** Baseline oncological characteristics of patients that underwent RAMIE between 04/2017 and 03/2022

	*N*	Percentage (%)
Patients	154	–
ASA I	36	23.4
ASA II	88	57.1
ASA III	30	19.5
ECOG 0	123	79.9
ECOG 1	26	16.9
ECOG 2	5	3.2
Pathology		
Adenocarcinoma	126	81.8
Squamous cell carcinoma	26	16.8
Other	2	1.3
Neoadjuvant treatment		
None	22	14.3
CROSS	75	48.7
FLOT	55	35.7
Other	2	1.3
Histopathological classification		
T0	25	16.2
T1a	11	7.1
T1b	23	14.9
T2	28	18.2
T3	61	39.6
T4	6	3.9
N0	89	57.8
N1	30	19.5
N2	17	11
N3	18	11.7
R0	146	94.8
R1	8	5.2

### Peri- and intraoperative course

In most patients, a robotic hybrid approach was applied, with the abdominal dissection, lymphadenectomy, and formation of the gastric conduit performed via conventional laparoscopy (*n* = 138; 89.6%), whereas the thoracic phase was performed robotic assisted in all patients [23]. Conversion of either the abdominal or thoracic phase was necessary in 11 patients (7%). In two patients, a bleeding occurred that was not manageable with a minimally invasive technique. In 9 patients, conversion was necessary due to oncological reasons (i.e., high intrathoracic tumor, large tumors with vessel adherence) to ensure oncological radicality. Mean duration of RAMIE was 371 min (range 217–615 min) overall, 422 min (range 340–615 min) in patients who underwent a fully robotic procedure, and significantly shorter with a mean of 366 min (range 217–579 min) in patients who underwent a laparoscopic abdominal phase combined with a robotic thoracic phase (*p* = 0.0013). Mean operating time for the robotically performed thoracic phase was 190 min (range 90–340 min). Mean operating time for the abdominal part including repositioning of the patient was 182 min (range 92–326 min).

### Postoperative course

The postoperative course and complications are summarized in Table 2. An anastomotic leak was seen in 24 patients (15.6%) of which 6 required surgical revision. The remaining patients received an interventional endoscopic treatment, i.e., endoscopic vacuum therapy.

**Table 2:** Postoperative course and summary of complications classified using the Clavien-Dindo classification after robotic-assisted esophagectomy

	*N* (Median)	Percentage (%)//range
Patients	154	–
Length of stay (days)	13	9–59
ICU (days)	2	1–112
Readmission to ICU	25	16.2
Postoperative complications		
CD 0	66	42.9
CD I	5	3.2
CD II	9	5.8
CD IIIA	47	30.5
CD IIIB	11	7.1
CD IVA	11	7.1
CD IVB	2	1.3
CD V	3	1.9
CCI	20.9	0–100

### Evaluation of performance levels

**Fig. 4:**
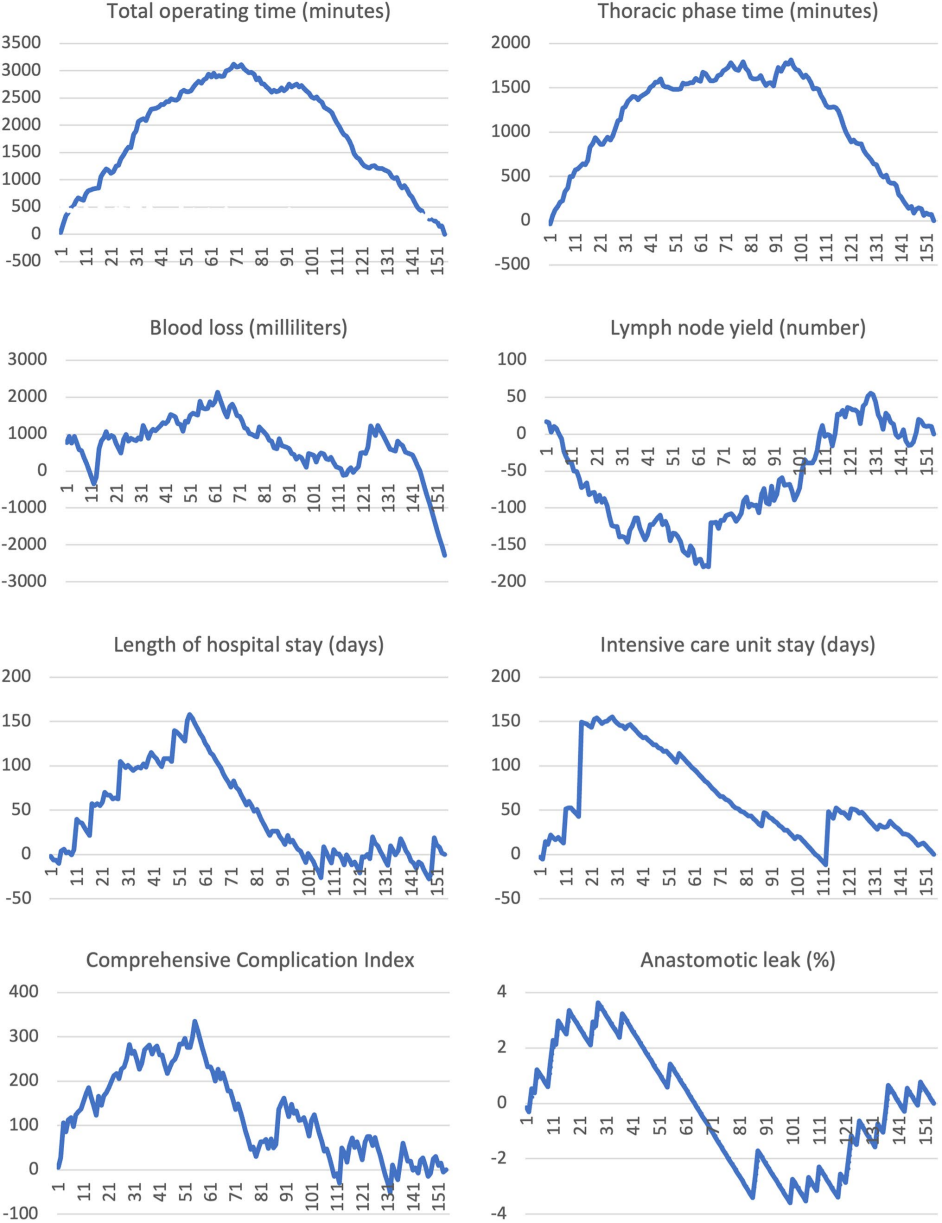
CUSUM analysis per variable

**Fig. 5:**
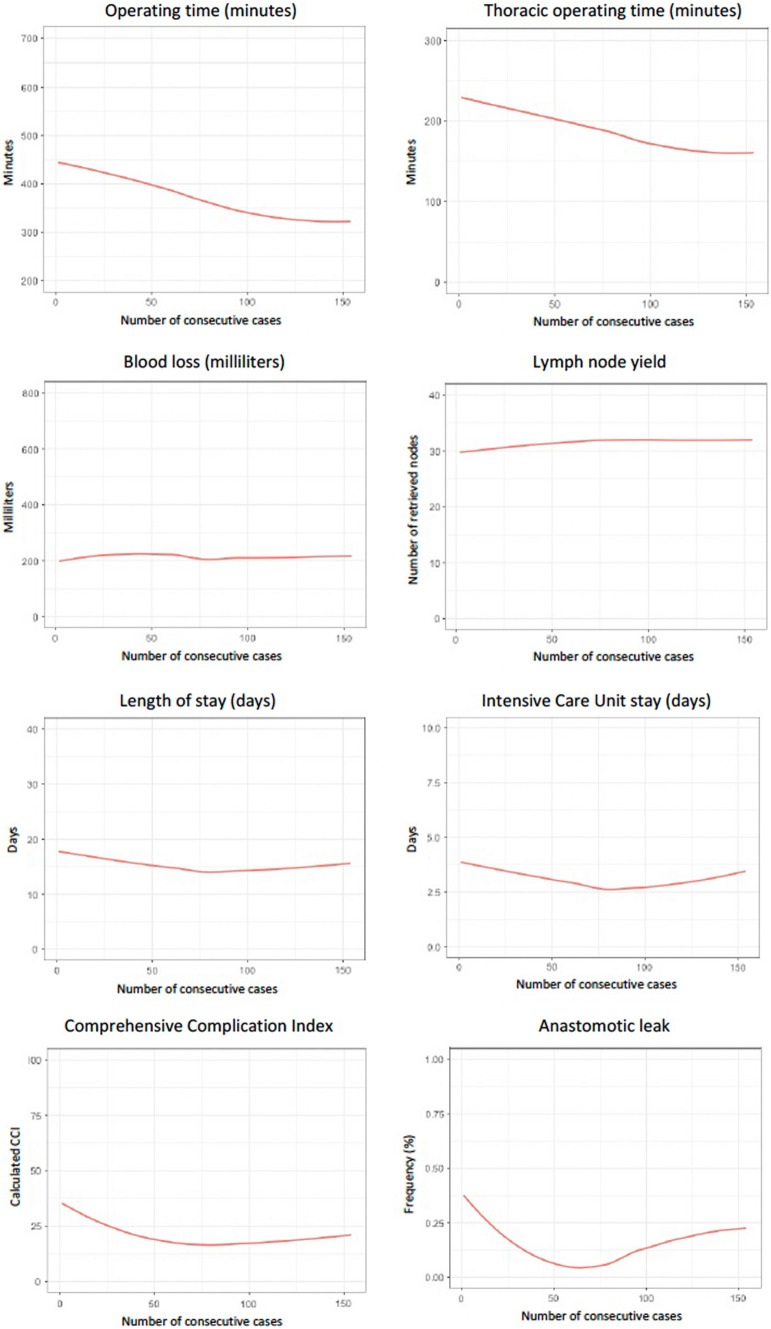
Fitted LOESS curves using generalized additive models

The reach of an expert performance level was assessed using both CUSUM techniques and LOESS curves. A visual assessment using linear regression was used to determine the inflection point where the linear regression line turns to zero [24]. CUSUM curves are presented in Fig. 4, LOESS curves are depicted in Fig. 5. An advancement in performance level was observed for operating time. For the total operating time, a clear inflection point was observed at case 70; when analyzing only the thoracic phase, the inflection point was at case 79.

For overall blood loss during surgery, the inflection point was at case 62, although it should be noted that no clear change for blood loss could be identified with the regression analysis. Lymph node yield showed an increase up until case 60 in the CUSUM analysis. This held also for regression; however, the model was less steep. No effect was observed for the rate of R0 resection. Overall R0 resection rate is shown in Table 1. Length of hospital stay stabilized after case 55. The overall length of stay on the intensive care unit stabilized at case 29. All days on ICU including postoperative surveillance and complication management were included in the analysis. All complications for each patient were graded according to the Clavien-Dindo classification, summarized in the comprehensive complication index (CCI). The CCI score inflection point was at case 55 in both CUSUM and regression analyses. Looking closer at the anastomotic leak rate, performance stabilized at case 38. At case 83, the inflection point started going upward again, a phenomenon seen as patient selection changed at that time, with more complex patients undergoing RAMIE including a complex comorbidity profile with especially cardiological and metabolic comorbidities, higher BMI, and older in age as well as more advanced tumors (T3, N+). This also holds for the regression analysis. An analysis of parameters in the first phase and the phase after the inflection point with corresponding *p*-values to evaluate statistical significance is shown in Table 3.

**Table 3:** Analysis of parameters pre- and post-inflection point as evaluated using the CUSUM method with corresponding p-values to evaluate the statistical significance

	Inflection point (case #)	Pre-inflection point	Post-inflection point	*p*-value
Operative time overall (min)	70	416	334	0.0001
Operative time thoracic phase (min)	79	214	167	0.0001
Blood loss (ml)	62	258	209	0.1043
R0 resection rate	NA	NA	NA	NA
Lymph node yield	60	34	39	0.0100
Length of hospital stay (days)	55	20	15	0.0006
Overall length of ICU stay (days)	29	10	4	0.0035
CCI Score	55	27	17	0.0119
Anastomotic leak (%)	38	23.7	12.9	0.1263

## Discussion

While previous studies have shown a detailed analysis of the learning curve for a variety of robotic surgeries, our study analyzes and helps understand the evolution of different performance levels and skill acquisition with the introduction of RAMIE at a high-volume center. The adoption of a modular step-up approach helped us introduce a new and complex surgical device into every-day clinical practice without compromises in patient safety, outcomes, and oncological quality of the resection by defining straightforward modules to divide a complex surgery into manageable steps [13]. Interestingly, no classic learning curve was seen as outcomes were not compromised despite the use of a new technique. At a competent performance level, we were able to define a standardized surgical technique using a circular stapling device and ICG for assessment of vascularization [5]. Reach of competency was seen in our ability to troubleshoot, as conversion rates dropped from 9.8% during earlier stages to 3% in cases performed since 2021. Following the steps of adult skill acquisition, proficiency and eventually expert performance levels were reached. A robotic training curriculum was defined based on the results of an international Delphi consensus study of leading experts for RAMIE and the results of this study [25, 26]. Using the cumulative sum method, our evolution and ascent to different performance levels were analyzed and the number of cases needed to overcome outcome measures compared to the expected mean was analyzed. It is worth noting that a different number of cases were needed for different outcome parameters. For operative time, 70 cases were needed to observe an inflection point, and 79 cases when solely looking at the thoracic phase. Only a slight difference in the number of cases was found, as the main skill acquisition, challenges to overcome, and technical difficulties were experienced during the thoracic phase of the operation. Furthermore, in 89.6% of cases, the abdominal phase was performed laparoscopically, not necessitating a new skill acquisition as this is the standard at our clinic. A previous study from Tagkalos et al. has shown that the true benefit of the robotic technique in oncological esophageal surgery probably lays in the thoracic part of RAMIE as enhanced visualization and precision of instruments leads to an improved thoracic lymphadenectomy [6]. It is noteworthy that the inflection points for the other parameters ranged from 29 cases for length of ICU stay to 60 for lymph node yield displaying how diverse the acquisition of skills sets and reach of individual performance levels can be. Furthermore, for the incidence of anastomotic leak, an interesting phenomenon was observed which correlated with clinical practice. At 38 cases, one inflection point was seen, illustrating the ascent in performance levels. At 83 cases, another inflection point was observed which correlated with a change in patient selection criteria at our clinic, as we began to perform RAMIE on more medically complex patients with a variety of preexisting comorbidities and locally advanced tumors, increasing the innate risk of anastomotic leak regardless of surgical technique.

Previous studies have shown the introduction of a robotic technique in oncological esophageal surgery and associated learning curves. A study from Yang et al. was able to show the effects of the learning curve on outcomes and surgical quality measures in their retrospective analysis of 400 patients that underwent a robotic-assisted minimally invasive McKeown procedure. Patients were divided in cohorts of *n* = 40 for the analysis. Combination and analysis of data showed an ascending, plateau, and descending phase, with the plateau phase being reached after 40 cases and the descending phase starting at a total of 215 cases. While only 40 cases were needed for a significant change in operating time, number of harvested lymph nodes, blood loss, and conversion rates, a total of 80 were needed to show a significant improvement in occurrence of anastomotic leak and vocal cord palsies [27]. Those findings correlate with our model of a performance levels, as Yang et al. probably observed an ascent between performance levels as their learning curve reached another descending phase. A recent study from Han et al. analyzed the learning curve of a single surgeon performing RAMIE. A retrospective analysis of the first 124 consecutive patients was performed using the risk-adjusted cumulative sum method [28]. However, while a careful analysis of data was performed, it must be noted that the surgical approach in the study changed after case 31 from a stapled to a robotically hand-sewn anastomosis, which may affect the postoperative outcomes and especially the shape of the learning curve. Results of the study were able to show that 51 cases are needed to overcome the learning curve for major complications and additional cases were required to see a decrease of operative time for the thoracic phase. As a conclusion, the authors postulate that there is an initial phase when starting the learning curve for a new procedure followed by a proficiency phase when the learning curve has been overcome, which may also be explained by the previously described skill acquisition model. A study from Egberts et al. showed the safe introduction of the RAMIE procedure with a standardized approach in a multicenter setting [29]. CUSUM analysis of the operative time showed significantly fewer procedures needed as seen in previous literature to overcome the learning curve with 22, 13, and 10 cases for different centers, respectively. Interestingly, a different learning curve was seen for different centers despite the introduction of a standardized technique illustrating the effect of each surgeon’s individual learning abilities and skill acquisition. Definition on the extent of the learning curve in this analysis was based on operative time. When comparing our outcomes of analysis to previously published data, it seems like our caseload is higher; however, it should be noted that we did not analyze the first learning curve but our way to reach proficiency.

Multiple analyses have divided cohorts into two or three groups according to the completion of the learning curve and reach of proficiency. While a vast variety of CUSUM analyses have shown different caseloads needed to overcome the learning curve for different parameters, studies still defined a specific number of procedures often based on a single parameter. Operative time is an objective parameter that is often used for assessment of the learning curve, while a retrospective analysis of a national database from Valsangkar et al. has shown that short and mid-long operative times do not correlate with postoperative outcome after Ivor Lewis esophagectomy [30]. Therefore, this parameter may not be accurate when defining a learning curve or change in performance level.

To reduce the number of cases needed to overcome the learning curve and reach a surgical level of proficiency, van der Sluis et al. have shown that a structured proctoring program is able to reduce the cases needed from 70 to 20 procedures [31]. However, a McKeown approach with a robotically hand-sewn cervical anastomosis was performed, limiting the comparability between this study and our data. In addition, measures of proficiency only included operating time, blood loss, and conversion rates, not considering postoperative outcomes and quality of the oncological resection. Nevertheless, we agree with the authors that a structured training curriculum is essential for maintaining patient safety and quality measures.

Many factors impact the learning curve, individual skill acquisition, and ascent in performance levels in a surgical setting; however, no validated definition yet exists. A review article from Kaul et al. concluded that in fact many end points including operative time, intra- and postoperative complications and outcome, conversion rates, blood loss, functional outcomes, and surgeon comfort with the procedure may provide the most accurate evaluation of the learning curve [32]. Previously published papers showed a variety of results and cases needed to overcome the learning curve and every study chose a different set of variables based on the author's choice. A meta-analysis from Pickering et al. summarizes the current evidence of learning curves for RAMIE with a range of 18–73 cases for lymph node yield and 20–80 cases for operative time, emphasizing that there is a need for standardization [33].

Additionally, surgical techniques especially during the introduction of a new complex technical device and patient selection differ significantly between centers. Limitations of our study include the retrospective study design and therefore limited availability of data. Furthermore, the learning curve of only two surgeons was analyzed limiting the transferability of the results to other surgeons and hospitals. In particular, our preexisting experience with surgery of the upper gastrointestinal tract including the application of different technical devices may have also influenced the results. Yet, based on our standardized technique, our large cohort of patients, and separate analysis of various factors, we believe that this study will help further understand the process of implementation of a new surgical device and robotic operating technique at a high-volume center. Furthermore, our previous experience with the implementation of new technical devices and diverse operative techniques in oncological surgery of the upper gastrointestinal tract makes this analysis unique.

Our data and analysis showed the progression from proficient to expert performance levels during the implementation of robotic-assisted minimally invasive Ivor Lewis esophagectomy at a European high-volume center and therefore complements previously published studies on the learning curve of RAMIE. The first introduction of the device and technique using a modular step-up approach allowed for a safe implantation without compromising patient safety or a classic learning curve. Further analysis of surgeons, especially with a different training status, has yet to reveal if the caseloads found in this study are universally applicable. However, skill acquisition and respective measures of such are diverse and as a great range of number of cases was observed, we believe that the learning curve and ascent in performance levels cannot be defined by one parameter alone.
